# Dosimetric evaluation of irradiation geometry and potential air gaps in an acrylic miniphantom used for external audit of absolute dose calibration for a hybrid 1.5 T MR‐linac system

**DOI:** 10.1002/acm2.13503

**Published:** 2021-12-16

**Authors:** Neelam Tyagi, Ergys Subashi, Dale Michael Lovelock, Stephen Kry, Paola Elisa Alvarez, Margie A Hunt, Seng Boh Lim

**Affiliations:** ^1^ Department of Medical Physics Memorial Sloan‐Kettering Cancer Center New York New York USA; ^2^ Department of Radiation Physics IROC MD Anderson Cancer Center Houston Texas USA

**Keywords:** 1.5 T magnetic field, electron return effect (ERE), IROC, MR‐guided radiation therapy, OSL

## Abstract

**Introduction:**

To investigate the impact of partial lateral scatter (LS), backscatter (BS) and presence of air gaps on optically stimulated luminescence dosimeter (OSLD) measurements in an acrylic miniphantom used for dosimetry audit on the 1.5 T magnetic resonance‐linear accelerator (MR‐linac) system.

**Methods:**

The following irradiation geometries were investigated using OSLDs, A26 MR/A12 MR ion chamber (IC), and Monaco Monte Carlo system: (a) IC/OSLD in an acrylic miniphantom (partial LS, partial BS), (b) IC/OSLD in a miniphantom placed on a solid water (SW) stack at a depth of 1.5 cm (partial LS, full BS), (c) IC/OSLD placed at a depth of 1.5 cm inside a 3 cm slab of SW/buildup (full LS, partial BS), and (d) IC/OSLD centered inside a 3 cm slab of SW/buildup at a depth of 1.5 cm placed on top of a SW stack (full LS, full BS). Average of two irradiated OSLDs with and without water was used at each setup. An air gap of 1 and 2 mm, mimicking presence of potential air gap around the OSLDs in the miniphantom geometry was also simulated. The calibration condition of the machine was 1 cGy/MU at SAD = 143.5 cm, *d* = 5 cm, G90, and 10 × 10 cm^2^.

**Results:**

The Monaco calculation (0.5% uncertainty and 1.0 mm voxel size) for the four setups at the measurement point were 108.2, 108.1, 109.4, and 110.0 cGy. The corresponding IC measurements were 109.0 ± 0.03, 109.5 ± 0.06, 110.2 ± 0.02, and 109.8 ± 0.03 cGy. Without water, OSLDs measurements were ∼10% higher than the expected. With added water to minimize air gaps, the measurements were significantly improved to within 2.2%. The dosimetric impacts of 1 and 2 mm air gaps were also verified with Monaco to be 13.3% and 27.9% higher, respectively, due to the electron return effect.

**Conclusions:**

A minimal amount of air around or within the OSLDs can cause measurement discrepancies of 10% or higher when placed in a high b‐field MR‐linac system. Care must be taken to eliminate the air from within and around the OSLD.

## 1 INTRODUCTION

Magnetic resonance (MR)‐guided radiation therapy (MRgRT) using a hybrid MR‐linear accelerator (linac) system have enabled real‐time guidance and online plan adaptation. One such system is the Unity 1.5 T (Elekta, Crawley, UK and Philips, Amsterdam, Netherlands) MR‐linac with a 7 MV Flattening Filter Free (FFF) Elekta linac and a high field strength 1.5 T Philips MR magnet.^1^ Higher field strength hybrid systems provide superior MR image quality for daily adaptation and therapy response assessment but pose multiple dosimetric challenges including reference dosimetry calibration.^2^ The impact of magnetic field on various chambers, dosimeters and measurement orientation on Unity has been well published.^2^, ^3^ The presence of small air gaps around an ion chamber (IC) or other dosimetry validation devices such as film or solid‐state detectors can cause over‐response in dosimetry measurement.^4^, ^5^ In addition to air, non‐standard geometry including extended source‐to‐axis (SAD) of 143.5 cm pose additional challenges for reference field dosimetry. Due to the presence of magnetic field and the extended SAD of the Unity, many users are adopting technical report series (TRS) 398 formalism (TPR(20,10) and 143.5 cm SAD, 5 cm depth) in order to avoid the uncertainties that arise from the extrapolation of TG‐51 parameters defined for conventional linacs. Tissue phantom ratio (TPR), as used in TRS 398, is also less affected by the b‐field than percent depth dose (PDD)s in the buildup region.

For external independent validation and audit of machine output, majority of US institutions participate in irradiation of NanoDots optically stimulated luminescence dosimeters (OSLDs; LANDAUER, Glenwood, IL, USA) embedded in a homogeneous acrylic block provided by Imaging and Radiation Oncology Core Quality Assurance Center in Houston (IROC).^6^ The acrylic block has been used to validate output on MRgRT systems as well.^7^, ^8^ These published studies investigated the feasibility of using IROC's acrylic block using thermoluminescent dosimeters (TLDs; ThermoFisher Scientific, Waltham, MA, USA). Wen et al. studied the directional dependence of single‐loaded TLD capsules using IROC's output verification acrylic TLD block on a 1.5 T Unity MR‐linac. They found that the TLD capsules with its long axis irradiated parallel to a 1.5 T magnetic field experienced a 2.3% higher output compared to TLD capsules with its long axis irradiated perpendicular to a 1.5 T magnetic field and attributed the likely cause to the inherent air gap within the single‐loaded TLD capsule itself. Steinmann et al.^7^ investigated double‐loaded TLDs with no air gap and found the output on Unity to be within 0.5% with respect to no magnetic field. These TLDs were irradiated in the acrylic block in full backscatter (BS) condition. One Unity user used TLDs provided by the University of Wisconsin Radiation Calibration Laboratory for their external output validation.^9^ The TLDs were housed in a cylindrical solid water (SW) holder with minimal air gaps which fit into the IC holder of their 1D water tank. The independent TLD validation was within 1% of their reported delivered dose. TLDs in above study was chosen as the dosimeter to perform independent validation as TLDs do not have a statistically significant response dependence with and without magnetic fields.

Considering the efficiency in the read‐out process,^6^ IROC replaced TLDs with OSLDs as their independent validation and audit of the machine output in 2010. The IROC acrylic block (i.e., miniphantom geometry) provides full electronic equilibrium but does not provide full photon lateral scatter (LS) or BS. The potential presence of small air gaps in and around the OSLDs may also impact the reading due to the electron return effect (ERE). Without the presence of magnetic fields, IROC's OSLD program has a measurement uncertainty of 2.8% (2 standard deviation, SD)^6^ which includes components such as calibration and readout and may potentially mask the measurement discrepancies when irradiated in a b‐field. In 2017, internal evaluation by IROC indicated the feasibility of using IROC acrylic blocks for output verification with OSLDs rather than TLDs for Unity MR‐linac machines.^10^ IROC conducted annual OSLD output monitoring using this program for several years with reasonable results. However, recently, IROC has observed results that showed unexpected results – doses much higher than expected – with their OSLD output program (personal communication). Therefore, in this study, we sought to investigate the response, and unexpected response, of the OSLD output measurements for the Unity standard geometry. The goal of this study is to investigate (a) the impact of side and BS on miniphantom geometry measurements and (b) the impact of potential air gaps on OSLD measurements on the Unity MR‐linac.

## 2 METHODS AND MATERIAL

### 2.1 Reference dose calibration using TRS 398

Absolute dosimetry on the in‐house Unity system was conducted following the TRS 398 formalism with the following calibration conditions: 10 × 10 cm^2^ field size, gantry angle 90° (left lateral or LL, for head‐first supine), 143.5 cm SAD, 5 cm depth, 1 cGy/MU.^11^ Reference dosimetry calibration was performed using an magnetic resonance imaging (MRI) compatible 1D water tank and an A12 MR (Standard Imaging, Middleton, WI, USA) Farmer‐type IC (sensitive volume = 0.64 cm^3^) with a valid Accredited dosimetry calibration laboratory (ADCL) calibration. The calibration process was done following the guidelines provided in publications by O'Brien et al.^2^ and Malkov and Rogers^12^ including the use of a chamber‐specific *k*_b_ factor that accounts for changes in chamber response due to the presence of magnetic field.^2^, ^12^ The beam quality, *k*_q_, was determined from TRS 398 using TPR(20,10). The measurement was performed with the long axis of the IC parallel to magnetic field, pointing out of the bore and gantry angle at 0° to remove any dependence due to varying helium level. Helium variation in the cryostat can result in up to 0.9% systematic difference between 0° gantry (anterior–posterior, AP) and 90° gantry (LL) orientation.^13^ Due to the extended SAD of Unity, the beam quality specifier measured was TPR(20,10).^11^

### 2.2 Experimental irradiation geometries

#### 2.2.1 IROC OSLD measurements

The external output validation was performed with the OSLDs and TLDs irradiated using a 10 × 10 cm^2^ field size, 100 MU, and gantry angle 0° (AP). The acrylic miniphantom block was placed on a thin IROC provided platform (which provides negligible BS). Since the Unity table height is fixed, the platform for the miniphantom geometry was adjusted so that the top of the platform was at an SAD of 143.5 cm (14 cm height above the table). The resulting nominal distance to the OSLD/TLD within the miniphantom was 142 cm (the OSLD/TLD are at 1.5 cm depth). Fourteen acrylic blocks with embedded OSLDs, and six sets of double‐loaded TLDs embedded in the acrylic miniphantom provided by IROC were irradiated in IROC miniphantom geometry between December 2019 and June 2021.

In addition to the IROC OSLD measurements, a spare acrylic miniphantom (without OSL embedded in it) from IROC was requested to do in‐house OSLD measurements. A replica of the IROC acrylic miniphantom was also built in‐house with an insert for a small A26 MR safe (sensitive volume = 0.015 cm^3^) IC. This allowed measurements to be done in the IROC geometry with an IC. The A26 chamber was cross‐calibrated with a Farmer‐type A12 MR chamber and was then used for absolute dose measurements as well as the experimental dose reference OSLDs were irradiated in parallel as well as perpendicular to the magnetic field direction (OSLD orientation shown in Section 2.2, Figure 2) while the A26 MR was irradiated only in the parallel orientation. It is well known that perpendicular irradiation of ICs in Unity MR‐linac can result in significant differences in dose compared to the parallel orientation.^12^, ^14^

#### 2.2.2 In‐house OSLD irradiation preparation

The details of in‐house OSLD preparation for irradiation at our institution are provided in a previous publication.^15^ We provide important steps here for completeness. The OSLD system was calibrated from 0 to 3000 cGy using TG‐191.^16^ All the in‐house OSLDs used in this study were taken from the same batch. To improve the dosimetric accuracy, each OSLD, *i*, was pre‐irradiated reference dose, *D*_ref_, of 50 cGy using a 6 MV beam on a conventional linac and was read out to determine the relative sensitivity of each detector (*k*_s,_ *_i_*). The *D*_ref_ of the irradiating machine was measured with the monthly IC and SW setup. The calibration factors of the monthly output setup were transferred from the annual TG‐51 calibration. A total of eight dots were taken from the group as reference and the corresponding reference measurement, *M*_ref_, was obtained by taking the average of these dots.

The impact of beam quality correction, *k*_q_, is typically small^16^ between 7 FFF and 6 MV and was taken to be 1.0. The linearity correction,^16^ *k*_L_, was determined by exposing the reference dots with an additional 100 cGy within 2 h of the irradiation at the Unity. Similar to the pre‐irradiation, the exposed dose, *D*_exp_, was measured with the monthly output setup. The *k*_L_ can be determined as:

$$k_{L}=\frac{D_{\exp}/M_{\exp}}{D_{\mathrm{ref}}/M_{\mathrm{ref}}}$$

As the time between the irradiation at the Unity and the reference machine is within 1 h, the effect of fading should be minimum^16^ and the corresponding correction, *k*_F_, was taken to be ∼1.0. The total dose given to each OSLD, *D_i_*, during measurements was then determined by correcting the net dose, *D*_o,_ *_i_*, as

$$D_{i}=D_{o,i}\cdot k_{s,i}\cdot k_{L}$$

#### 2.2.3 Experimental in‐house OSLD irradiation conditions

**FIGURE 1:**
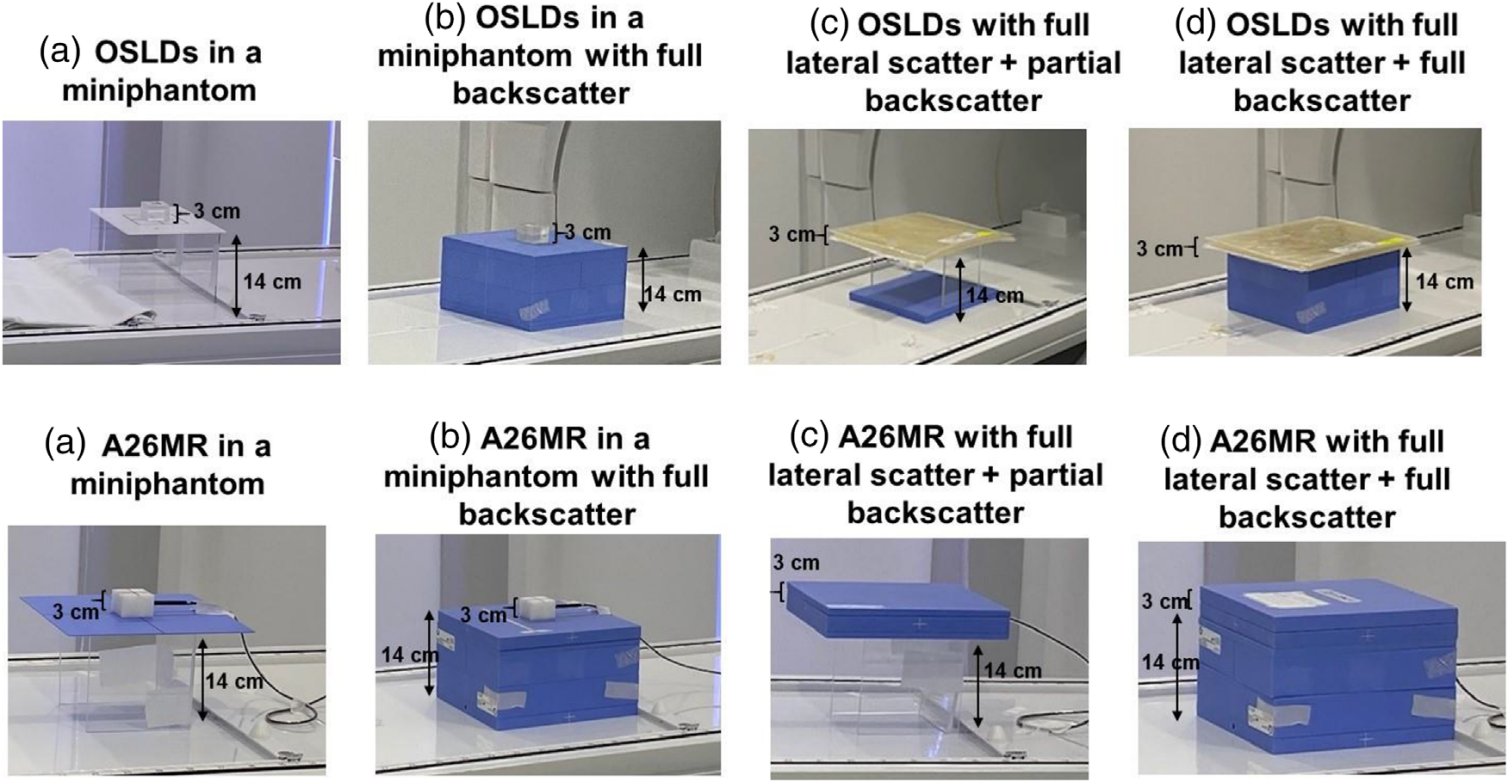
Optically stimulated luminescence dosimeter (OSLD) and A26 magnetic resonance (MR) ion chamber in various irradiation geometries. Miniphantom platform or solid water stack at 143.5 cm SAD (or 14 cm table height)

The following irradiation geometries were used for in‐house measurements on the Unity (Figure 1).

1. Miniphantom irradiation in IROC geometry with partial LS and partial BS (Figure 1a).
2. IC/OSLD in a miniphantom placed on a SW stack (25 × 25 × 14 cm^3^) at a depth of 1.5 cm (partial LS + full BS) (Figure 1b).
3. IC/OSLD placed at a depth of 1.5 cm centered inside a 3 cm slab of SW or 3 cm buildup (superflab) material (full phantom with full LS + partial BS) (Figure 1c).
4. IC/OSLD centered inside a 3 cm slab of SW or buildup material at a depth of 1.5 cm placed on top of a SW stack phantom (full phantom with full LS + full BS) (Figure 1d).

A computed tomography (CT) scan of the miniphantom with OSLDs (acquisition voxel size = 0.9 × 0.9 × 0.9 mm^3^) showed a slight air gap above the OSLDs. Hence, another set of OSLD measurements was performed by injecting water into the OSLD cassette in an effort to surround the active volume in water. A 14‐guage needle was used to force the air out of the OSLD cassettes. Once the water was injected and OSLDs irradiated, compressed air was used to push out all the water to dry out as much as possible. The OSLDs were left out overnight before reading them. No heat or any form of thermal process was used to dry the OSLs. Two OSLDs were irradiated for each setup, with and without water and in parallel and perpendicular orientations. An average reading between the two OSLDs was reported. The delivery was repeated, 2 months apart to obtain a spread in OSLD measurements. A total of 64 in‐house OSLDs were irradiated.

**FIGURE 2:**
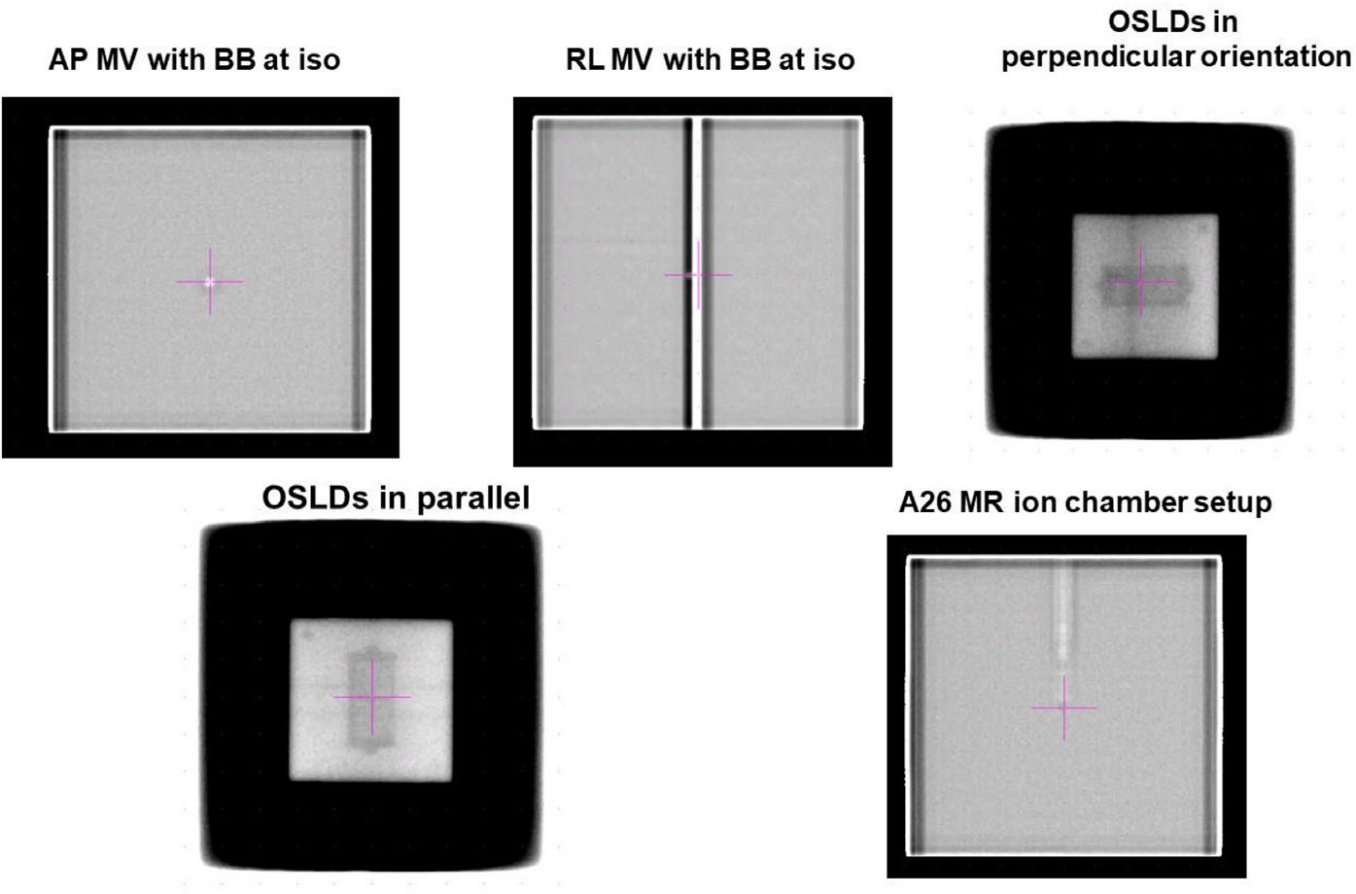
Image‐guided setup of Imaging and Radiation Oncology Core Quality Assurance Center (IROC) miniphantom with optically stimulated luminescence dosimeters (OSLDs) in parallel and perpendicular (to magnetic field direction) configuration using onboard MV imager and a metal BB. Also shown is the setup image of the A26 magnetic resonance (MR) ion chamber

Lack of lasers in the Unity room necessitated an image‐guided setup and verification of irradiation geometry using an onboard MV imaging panel. Figure 2 shows example MV images of OSLDs and IC. Before the placement of OSLD block, a metal BB was placed at the isocenter and its location was confirmed with the MV images at 0° (AP) and 90° (LL). Once the isocenter position was established, the BB was replaced with the OSLD block.

#### 2.2.4 A26 MR cross‐calibration process

Absolute dose, *D*_A12_(5), was first measured with an A12 MR IC at the depth of 5.0 cm, SAD = 143.5 cm, field size of 10 × 10 cm^2^, and 100 MU in SW which is traceable to reference dosimetry in water using TRS 398. The bias voltage of the electrometer was set at +300 V for the A26 MR for all the cross‐calibration and measurements. The A12‐MR was then replaced with an A26 MR IC in the same setup condition and similar 100 MUs were given to the A26 setup to obtain the measurements, *M*_A26_(5). As measurements were done with the same Max4000 (Standard Imaging) electrometer, a cross‐calibration factor, *k*_A26_, was determined with the following relationship:

$$k_{A26}=\frac{D_{A12}\left(5\right)}{M_{A26}\left(5\right)P_{\mathrm{elec}}k_{\mathrm{TP}}}$$

where *P*_elec_ and *k*_TP_ are the electrometer calibration factor, and the temperature and pressure correction factor, respectively. As the bias voltage was maintained to be consistent to be the same during cross‐calibration and measurements, the ion recombination and polarity effects of A26 were implicitly incorporated in *k*_A26_. With this *k*_A26_, A26 could be used to measure absolute dose, *D*_A26_, with the following expression:

$$D_{A26}=k_{A26}P_{\mathrm{elec}}k_{\mathrm{TP}}M_{A26}$$

With this relationship established, the OSLD measurements were repeated with the A26 MR. Measurements were performed with the chamber in parallel orientation. Before inserting the chamber in the miniphantom, the slot was filled with water so that any potential air surrounding the chamber's sensitive volume could be removed. The four setups as shown in Figure 1 were also used to irradiate the acrylic miniphantom with an A26 MR insert.

### 2.3 Monaco Monte Carlo simulations of irradiated geometries

The four irradiation geometries described in Figure 1 were also recreated in Monaco™ treatment planning system (TPS) to facilitate Monte Carlo simulations and dose calculations. Graphics processing unit Monte Carlo dose (GPUMCD) calculations were performed with a voxel size of 1 × 1 × 1 mm^3^ and a statistical uncertainty of 0.5% and point dose was measured at the OSLD measurement depth of 1.5 cm along the central axis. Additional calculations were done by simulating a uniform air gap of 1 and 2 mm at the OSLD measurement point in the acrylic miniphantom geometry to assess the impact of ERE due to a potential air gap. Finally, acrylic miniphantom with OSLDs in place was also CT scanned and Monaco calculations were performed with OSLDs in both parallel and perpendicular configurations. Monaco calculations were done as is without overriding the electron density in the OSLD slot.

## 3 RESULTS

### 3.1 Reference dosimetry

**FIGURE 3:**
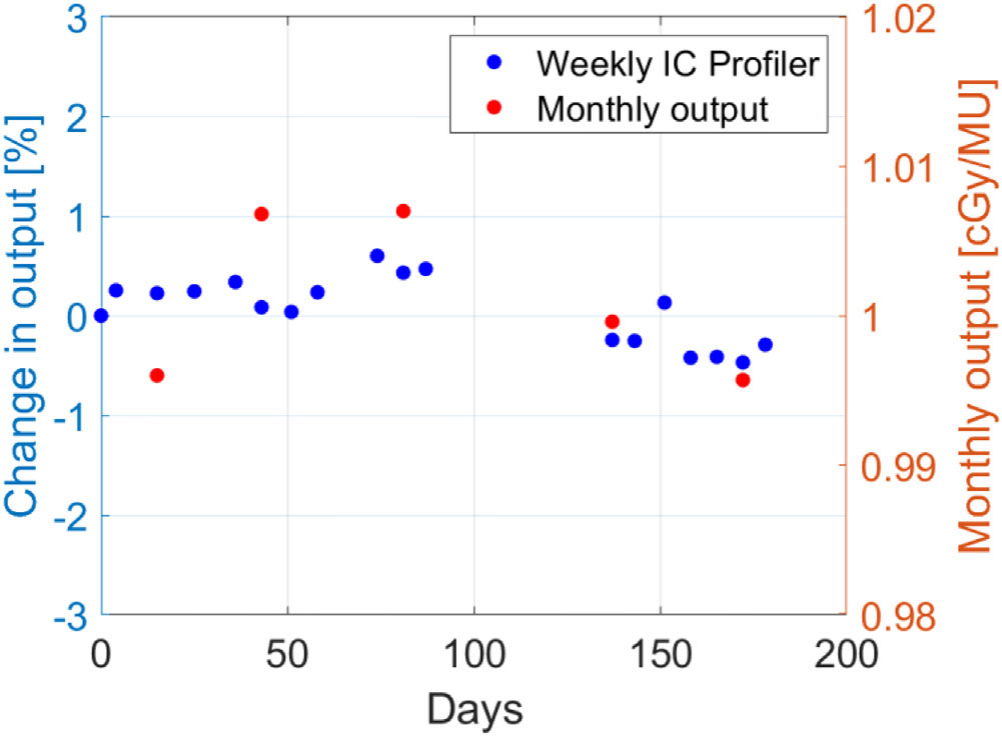
Machine output on Unity using weekly ion chamber (IC) profiler (left axis) and monthly A12 magnetic resonance (MR) (right axis) output in solid water.

The TPR(20,10) beam quality specifier measured at gantry 0° (AP) was 0.708. Using TRS 398, the reference output was set to 1.000 cGy/MU at initial absolute dose calibration. Monthly output measurement using the same A12 MR IC but in SW geometry was setup based on TRS 483 formalism.^17^ The monthly output trend in SW as well as weekly IC measurement using the IC profiler are shown in Figure 3. Machine stability measured over a period of the first 200 days of clinical operation using IC profiler was 0.053 ± 0.33% with respect to the baseline reference value. Absolute dose stability measured with A12 MR was 1.001 ± 0.006 cGy/MU. Independent output measurements by IROC with TLDs with respect to our institution output was 1.007 averaged over six measurements (SD 0.009). Independent output measurements by IROC with OSLDs (average of 14 irradiations) with respect to our institution output was 1.044, ranging from 0.989 to 1.094 and with a SD of 0.032. The OSLD approach showed both a bias for overestimating the output and a much larger SD.

### 3.2 Monte Carlo simulations

**FIGURE 4:**
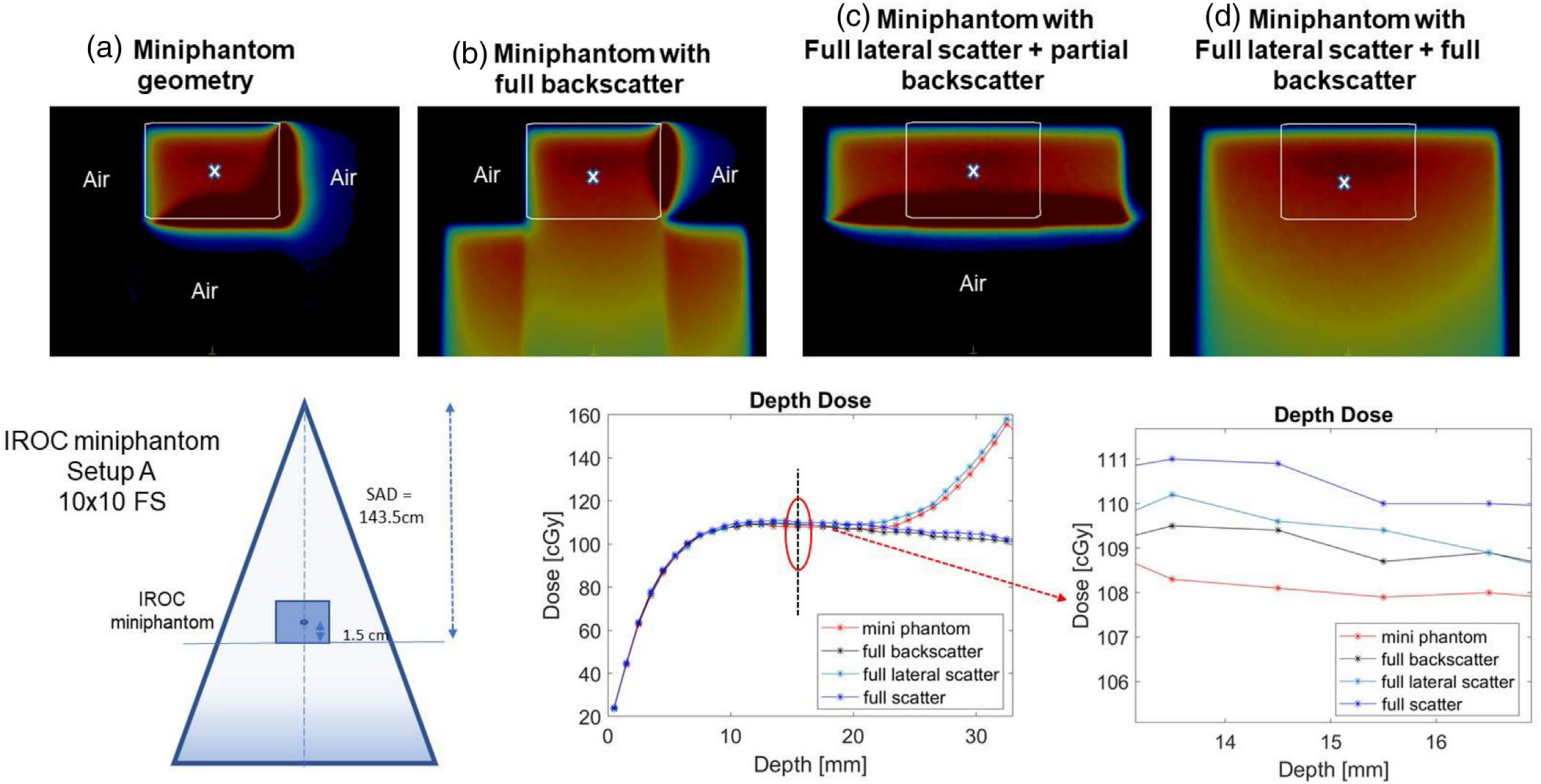
Monte Carlo simulations for different optically stimulated luminescence dosimeter (OSLD) irradiation geometries. Cross represents the measurement point as well as the point through which the central axis depth dose profile is extracted

Figure 4 shows the OSLD calculation geometry. The expected dose at the OSLD measurement point based on the machine calibration condition should be $D_{0}\left(10\times 10\;cm^{2},\;143.5\;\mathrm{cm}\;\mathrm{SAD},5\;\mathrm{cm}\;\mathrm{depth}\right)/\left(\mathrm{TMR}\times \mathrm{ISF}\right)$, where ISF (= 0.983) is inverse square factor, tissue maximum ratio (TMR) = 0.926. The expected dose is also shown in Figure 4. Ignoring any scatter differences due to the dimensions of the phantom, the expected dose at the OSLDs was 110 cGy. Figure 4 also shows an axial dose color wash through the central axis plane. The cross represents the OSLD dose measurement point used in Tables 1 and 2. The depth dose curve also passes through this point. The dose values calculated at the OSLD measurement point using Monte Carlo for the four setups (acrylic miniphantom, partial LS + full BS, full LS + partial BS, full LS and BS) are 108.2, 108.1, 109.4, and 110.0 cGy, respectively. Please note that each point dose has a statistical uncertainty of 0.5%. These differences are expected because of the differences in scatter conditions. The missing scatter factors for this block is 2.9% in a standard 6 MV beam to account for the difference between the miniphantom geometry and full phantom conditions; this expected value compares well with the measured difference of 2.8%.

**TABLE 1:** Dosimetric results (parallel orientation): comparison between optically stimulated luminescence dosimeter (OSLD), ion chamber, and Monaco for four different configurations

Measurement configurations	OSLD (with water)	OSLD (without water)	IROC OSLD (without water)	A26 MR output (gold standard)	Monaco^a^
Miniphantom geometry	109.1	123.8	111.6 ± 3.2	109.0 ± 0.03	108.2
Miniphantom + full backscatter	112.0	125.3	–	109.5 ± 0.06	108.1
Full phantom + partial backscatter	107.6	125.8	–	110.2 ± 0.02	109.4
Full phantom + full backscatter	111.9	124.0	–	109.8 ± 0.03	110.0

*Note*: Measurements from Imaging and Radiation Oncology Core Quality Assurance Center (IROC) provided OSLDs in the miniphantom geometry at reference depth are also included here (third column). Measurement depth is the depth of OSLD/ ion chamber (IC) placement in the miniphantom = 1.5 cm.

Abbreviation: MR, magnetic resonance.

^a^ Monaco simulations performed with 0.5% statistical uncertainty.

**TABLE 2:** Dosimetric results (perpendicular orientation): comparison between optically stimulated luminescence dosimeter (OSLD), ion chamber, and Monaco for four different configurations

Measurement configurations	OSLD (with water)	OSLD (without water)	A26 MR^a^ (gold standard)	Monaco
Miniphantom geometry	109.5	119.0	–	108.2
Miniphantom + full backscatter	112.2	125.4	–	108.1
Full phantom + partial backscatter	112.7	123.3	–	109.4
Full phantom + full backscatter	112.8	121.6	–	110.0

^a^ A26 magnetic resonance (MR) measurements are not performed in perpendicular orientation.

**FIGURE 5:**
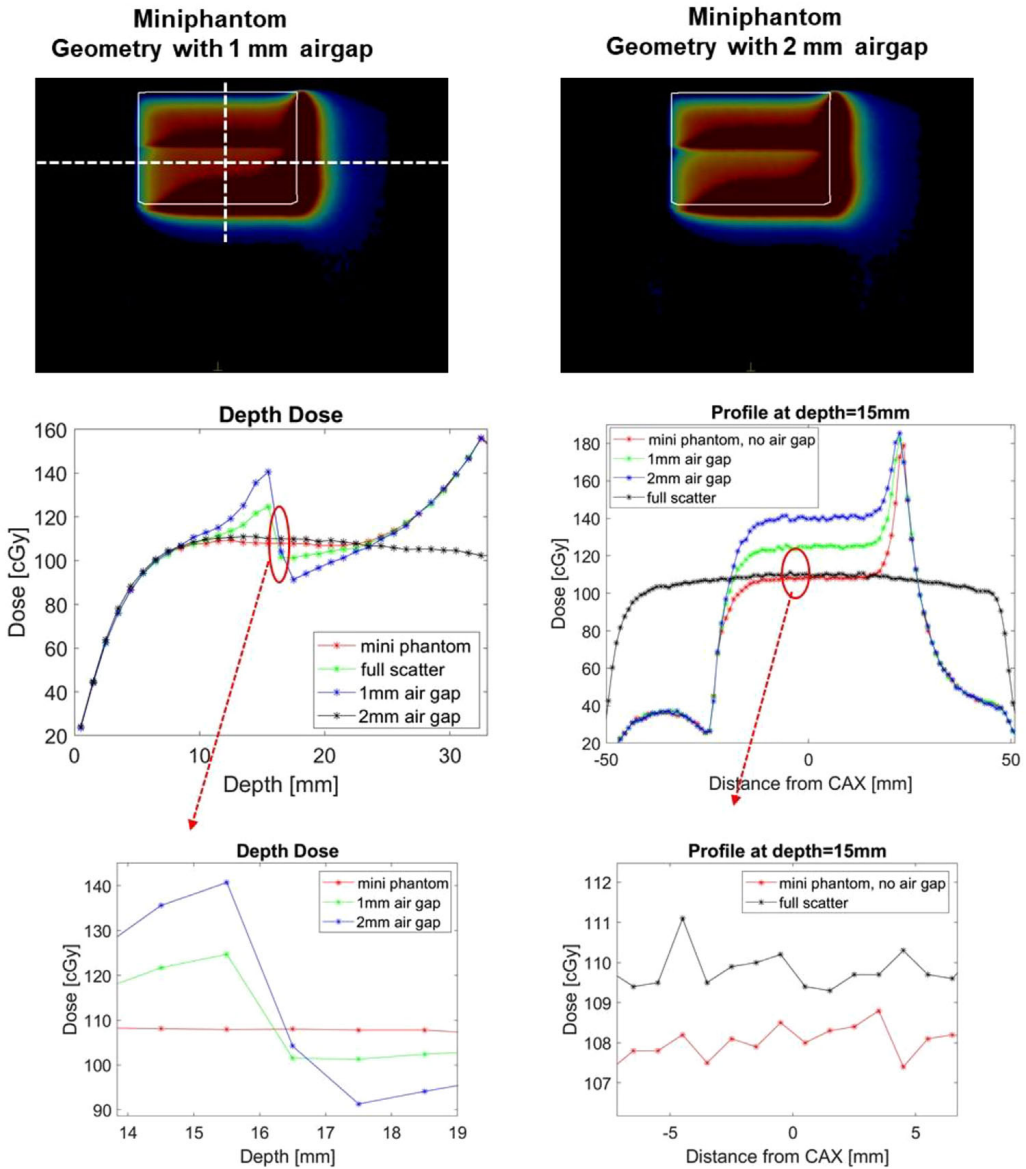
Miniphantom geometry simulating 1 and 2 mm air gap near the placement of optically stimulated luminescence dosimeters (OSLDs) (top). Depth dose curves through the center of the phantom and profiles at the OSLD measurement point (bottom). For clarity, insets show a magnified region around the OSLD measurement point

An increasing air gap of 1 or 2 mm at the measurement depth (mimicking presence of potential air gap around the OSLDs) can cause additional ERE and increasing dose enhancement at the measurement point. The dose with a 1 or 2 mm air gap at the OSLD measurement point was 124.7 and 140.7 cGy, respectively (Figure 5).

**FIGURE 6:**
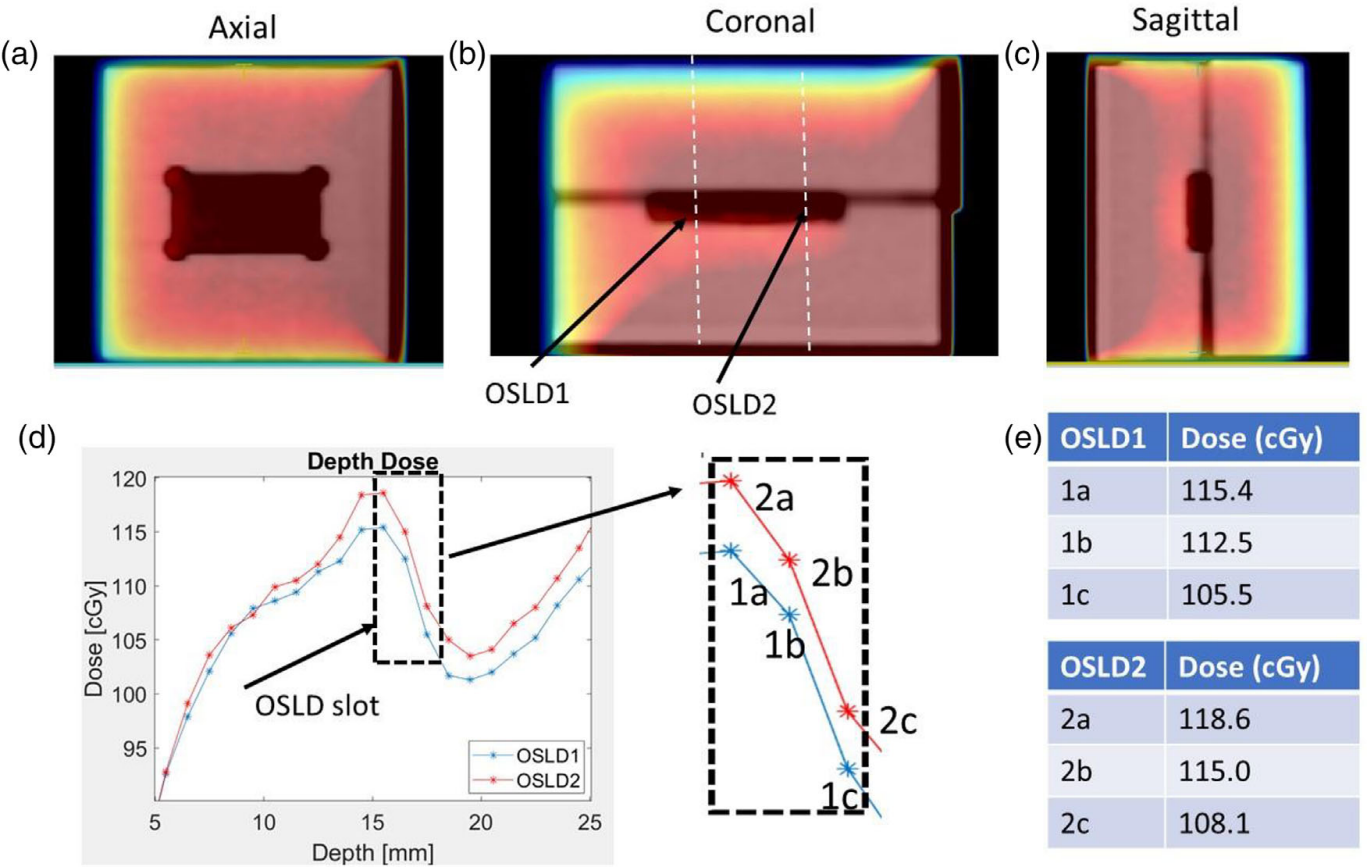
Monte Carlo dose simulations using computed tomography (CT) scan of acrylic miniphantom. (a–c) Axial, coronal, and sagittal dose color wash of the scanned phantom. (d) Depth doses are calculated through the center of each optically stimulated luminescence dosimeter (OSLD) in the phantom. Dashed lines in the coronal plane represents the plane through which depth doses were extracted. (e) Dose measurements at the calculation points within the OSLD slot. Monte Carlo calculations were performed without overriding the electron density

Calculations were also done in the CT scanned miniphantom with two OSLDs in place. Figure 6 shows the scanned phantom, Monaco TPS dose distribution as well profiles though the center of both OSLDs were extracted. Monaco calculations were done as is without overriding the electron density in the OSLD slot. Both OSLDs are in the gradient region because of the air gap surrounding them in the slot. The dose measurements at the calculation points within slot were 115.4, 112.5, and 105.5 cGy for OSLD1 and 118.6, 115, and 108.1 cGy for OSLD2, respectively.

### 3.3 A26 IC and OSLD measurements

The IC measurements in the four configurations were 109.0 ± 0.03, 109.5 ± 0.06, 110.2 ± 0.02, and 109.8 ± 0.03 cGy, respectively. In‐house OSLD measurements were performed both with and without the water. Without water, OSLDs consistently read more than 10% high dose compared to the expected value of 110 cGy. Once water was injected inside and outside the OSLDs the measurement values were 112.0, 112.0, 107.6, and 111.9 cGy, respectively, in parallel orientation. The corresponding values in perpendicular configuration were 112.8, 112.2, 112.7, and 112.8 cGy, respectively. The SD in OSLD measurement with water was 2.4%. The SD in OSLD measurement without water was 1.1%. Tables 1 and 2 summarize the results from Monaco calculations, A26 MR chamber measurements, and OSLD measurements in the four different setup geometries. Table 1 also includes the average result of 14 IROC OSLD irradiations embedded in the IROC acrylic miniphantom and read at the detector location at IROC QA center.

## 4 DISCUSSION

In this study, we investigated the feasibility of using an acrylic miniphantom and other geometries for dosimetry audit and the presence of air gaps in the miniphantom geometry on the output measurement for Unity 1.5 T MR‐linac system. Monte Carlo simulations showed that in the absence of full LS and BS material, the dose distribution shifts laterally due to ERE. ERE also results in electron curling at the back of the phantom material with an acrylic–air interface, resulting in dose deposition at the posterior part of the phantom (Figure 4a). Once the water‐equivalent BS material was added, only a lateral shift in profile was seen due to ERE (Figure 4b). In the presence of full LS conditions and partial BS, the lateral profile shift was no longer observed (Figure 4d). The OSLD measurement point remained far from the influence of ERE in the acrylic miniphantom geometry. Calculations in full scatter conditions were 2.1% higher compared to acrylic miniphantom geometry. A difference of >10% in machine output was measured and calculated when a 1 mm of air was introduced around the OSLD measurement point.

Calculations done with a CT scanned acrylic phantom showed similar behavior. Monaco calculations were done as is without overriding the electron density in the OSLD slot. Because of a slight air gap around the OSLDs, the calculation is comparable to the simulation for which a 1 mm air gap was used. There is also a big gradient with high dose uncertainty where the OSLDs are placed. Please note that the electron density in each voxel in Monaco is calculated based on the weighted average of the material that occupies the voxel. In the air slot containing the OSLDs, the electron density varied widely from ∼0.1 to ∼0.9, depending on the relative voxel occupation of the phantom, OSLD material, and air. The resulting dose gradient in this case is decreased due to partial volume effects and would be much larger in the absence of averaging. The minimum grid size in Monaco is currently 1 mm. TPS dose calculation limitations related to a minimum allowed voxel size of 1 mm^3^ as well as cut‐off energy of 189 keV for secondary electron transport within GPUMCD Monte Carlo calculation in Monaco further warrants the use of more rigorous Monte Carlo calculations with sub‐millimeter voxel size and lower secondary electron cut‐off energy in determining whether Monaco is overestimating the ERE contribution in the presence of air gap. Monte Carlo modeling of the OSLDs sensitive volume, cassette, sub‐millimeter air gaps inside the OSLDs and potential air gaps between the OSLDs and miniphantom would further help in understanding the discrepancy.

IC measurements consistently showed ∼110 cGy in all four configurations which is also the expected dose at this point. OSLs placed in perpendicular orientation measured systematically higher dose as compared to parallel orientation although the dose values are within OSL measurement uncertainty. If possible, parallel orientation/placement of the chips is recommended to minimize any potential discrepancy that may arise due to ERE. BS factor was also not significant in IC or OSLD measurements provided the measurement depth stays consistent. Beyond 2 cm depth in the acrylic miniphantom, ERE on the exit surface becomes quite significant.

**FIGURE 7:**
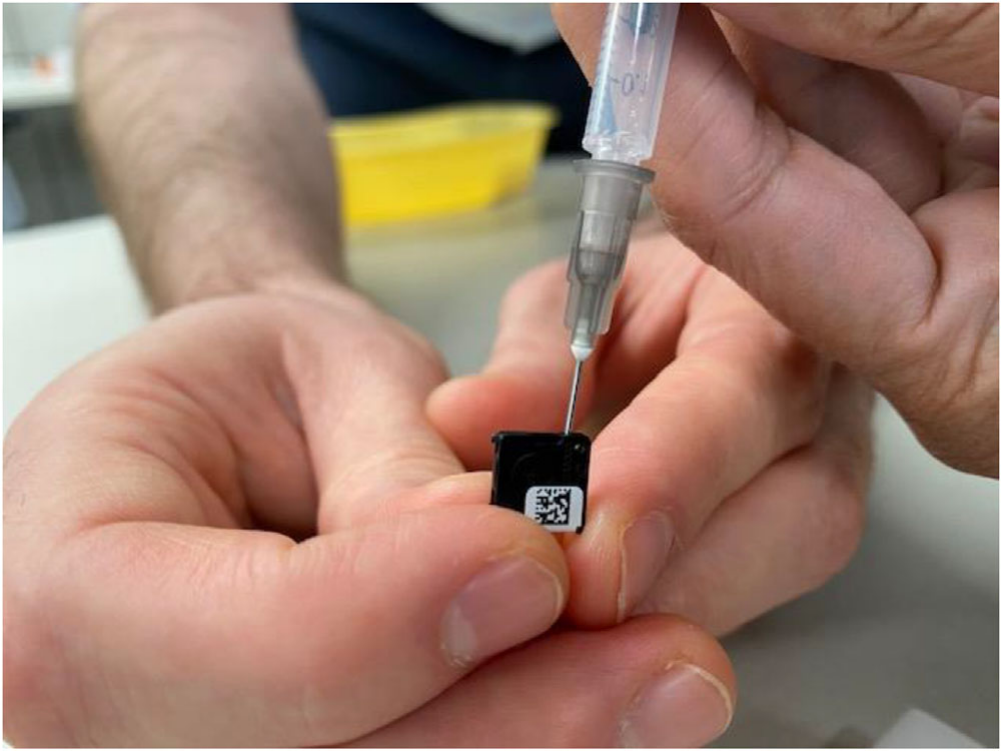
Using a 14‐gauge needle to inject water within the optically stimulated luminescence dosimeters (OSLDs) to force air out. Compressed air must be used to dry before reading the OSLDs

OSLD measurements with and without water showed >10% difference in dose values. The process of placing OSLD in water is suggested to be safe for these detectors as per AAPM TG‐191 report.^16^ We also noticed that simply floating the OSLDs in water was not enough. Our first round of experiments showed OSLD readings similar to the ones without any water implying that the air trapped inside the OSLs could also contribute to ERE. We then resorted to injecting water inside the OSLs with a syringe to force the air out. Figure 7 shows the 14‐gauge needle that was used to force the air out. Please note that we did not use heat or any form of thermal process to dry the OSLs. The variation in OSLD reading with water also shows that (a) the air gap within (or around) the OSLD is variable and (b) our water injection method is not perfect and needs further investigation. A recent study published by Kim et al.^18^ used OSLDs for surface dose measurements. The authors confirm that there are sub‐millimeter air gaps in the construction of the OSLD that may alter the dose response in the presence of high b‐field. However, the authors found the air gap effect within the OSLDs on dose measurements to be likely minimal in their study. The authors also immersed their radiation exposed OSLDs in water and found the dose readings to be lower indicating that perhaps the trapped electrons are perturbed by contact with water. In our study, we immersed or injected the OSLDs with water first and then irradiated to avoid the effect if ERE due to air.

Our analysis shows that if there are large manufacturing tolerances in the placement of OSLDs within the acrylic block, for example, depth, air around or within the dots, the OSLD output measurements can be severely impacted and may result in a higher output discrepancy compared to institutional standard. We recommend that institutions starting their MR‐guided program order both OSLDs and TLDs for an independent audit of their reference dosimetry to understand any potential discrepancy, if any, in output measurements with OSLDs.

## 5 CONCLUSIONS

Our investigation shows that a minimal amount of air around or within the OSLD packets in the acrylic miniphantom geometry can cause output discrepancies of the order of 10% or higher when placed in a high b‐field 1.5 T Unity MR‐linac system. Care must be taken by groups performing measurements in high b‐field with OSLDs to eliminate the air from within and around the OSLD. In the event that significant output discrepancies are observed during dosimetry audits, the possibility of inaccurate results due to the presence of air gaps must be thoroughly investigated prior to changes to the Unity absolute dose calibration.

## CONFLICT OF INTEREST

None.

## AUTHOR CONTRIBUTIONS

Neelam Tyagi, Ergys Subashi, and Seng Boh Lim conceptualized the study with the help of Dale Michael Lovelock and Margie A Hunt. Neelam Tyagi, Ergys Subashi, and Seng Boh Lim performed all the institutional measurements, performed dose calculations and data analysis. Stephen Kry and Paola Elisa Alvarez provided results and support for IROC OSLD measurements. Neelam Tyagi, Ergys Subashi, and Seng Boh Lim wrote the manuscript. Dale Michael Lovelock, Margie A Hunt, Stephen Kry, and Paola Elisa Alvarez contributed in manuscript editing.

## Supporting information

Supporting Information
