# Supplementary material for: Dosimetric evaluation of irradiation geometry and potential air gaps in an acrylic miniphantom used for external audit of absolute dose calibration for a hybrid 1.5 T MR‐linac system
Source: J Appl Clin Med Phys. 2021 Dec 16;23(2):e13503. doi: 10.1002/acm2.13503 (PMC8833292; doi:10.1002/acm2.13503)
Supplement: Supplementary file 1 — Supporting Information [file ACM2-23-e13503-s001.docx]

**Dosimetric evaluation of irradiation geometry and potential air gaps in an acrylic miniphantom used for external audit of absolute dose calibration for a hybrid 1.5T MR-linac system**

*^1^Neelam Tyagi, *^1^Ergys Subashi, ^1^Dale Michael lovelock, ^2^Stephen Kry, ^2^Paola Elisa Alvarez, Margie A Hunt and ^1^Seng Boh Lim

*Co-first authors

^1^Department of Medical Physics, Memorial Sloan-Kettering Cancer Center, New York, NY 10065, USA

^2^Department of Radiation Physics, IROC, MD Anderson Cancer Center, Houston, Tx 77030, USA

Corresponding author:

Neelam Tyagi, PhD

Department of Medical Physics,

Memorial Sloan Kettering Cancer Center

545 74^th^ street, New York, NY 10065

Tel: (646) 608-2479

E-mail: tyagin@mskcc.org

**Running Title**: Dosimetric evaluation in an acrylic Miniphantom for MRgRT

**Key words:** IROC, OSL**,** Electron Return Effect (ERE), 1.5 T magnetic field, MR-guided radiation therapy

**Acknowledgements**

NT, ES and SL conceptualized the study with the help of DL and MH. NT, ES and SL performed all the institutional measurements, performed dose calculations and data analysis. SF and PA provided results and support for IROC OSLD measurements. NT, ES and SL wrote the manuscript and DL, MH, SF and PA contributed in manuscript editing.

This research was partially supported by the NIH/NCI Cancer Center Support Grant/Core Grant (P30 CA008748) and support grant CA180803. Authors are grateful to Dr Eric Paulson for discussion on OSLD measurements in a miniphantom.
